# Comparing Digital to Conventional Physical Therapy for Chronic Shoulder Pain: Randomized Controlled Trial

**DOI:** 10.2196/49236

**Published:** 2023-08-18

**Authors:** Sang S Pak, Dora Janela, Nina Freitas, Fabíola Costa, Robert Moulder, Maria Molinos, Anabela C Areias, Virgílio Bento, Steven P Cohen, Vijay Yanamadala, Richard B Souza, Fernando Dias Correia

**Affiliations:** 1 Department of Physical Therapy and Rehabilitation University of California San Francisco San Francisco, CA United States; 2 Sword Health Inc Draper, UT United States; 3 Institute of Cognitive Science University of Colorado Boulder Boulder, CO United States; 4 Department of Physical Medicine & Rehabilitation Johns Hopkins School of Medicine Baltimore, MD United States; 5 Department of Anesthesiology and Physical Medicine and Rehabilitation and Anesthesiology Uniformed Services University of the Health Sciences Bethesda, MD United States; 6 Department of Neurology Centro Hospitalar e Universitário do Porto Porto Portugal

**Keywords:** chronic shoulder, clinical outcome, digital care, digital health intervention, musculoskeletal pain, pain management, physical therapy, remote sensing technology, telerehabilitation

## Abstract

**Background:**

Chronic shoulder pain (CSP) is a common condition with various etiologies, including rotator cuff disorders, adhesive capsulitis, shoulder instability, and shoulder arthritis. It is associated with substantial disability and psychological distress, resulting in poor productivity and quality of life. Physical therapy constitutes the mainstay treatment for CSP, but several barriers exist in accessing care. In recent years, telerehabilitation has gained momentum as a potential solution to overcome such barriers. It has shown numerous benefits, including improving access and convenience, promoting patient adherence, and reducing costs. However, to date, no previous randomized controlled trial has compared fully remote digital physical therapy to in-person rehabilitation for nonoperative CSP.

**Objective:**

The aim of this study is to compare clinical outcomes between digital physical therapy and conventional in-person physical therapy in patients with CSP.

**Methods:**

We conducted a single-center, parallel-group, randomized controlled trial involving 82 patients with CSP referred for outpatient physical therapy. Participants were randomized into digital or conventional physical therapy (8-week interventions). The digital intervention consisted of home exercise, education, and cognitive behavioral therapy (CBT), using a device with movement digitalization for biofeedback and asynchronous physical therapist monitoring through a cloud-based portal. The conventional group received in-person physical therapy, including exercises, manual therapy, education, and CBT. The primary outcome was the change (baseline to 8 weeks) in function and symptoms using the short-form of Disabilities of the Arm, Shoulder, and Hand questionnaire. Secondary outcome measures included self-reported pain, surgery intent, analgesic intake, mental health, engagement, and satisfaction. All questionnaires were delivered electronically.

**Results:**

A total of 90 participants were randomized into digital or conventional physical therapy, with 82 receiving the allocated intervention. Both groups experienced significant improvements in function measured by the short-form of the Disabilities of the Arm, Shoulder, and Hand questionnaire, with no differences between groups (–1.8, 95% CI –13.5 to 9.8; *P*=.75). For secondary outcomes, no differences were observed in surgery intent, analgesic intake, and mental health or worst pain. Higher reductions were observed in average and least pain in the conventional group, which, given the small effect sizes (least pain 0.15 and average pain 0.16), are unlikely to be clinically meaningful. High adherence and satisfaction were observed in both groups, with no adverse events.

**Conclusions:**

This study shows that fully remote digital programs can be viable care delivery models for CSP given their scalability and effectiveness, assessed through comparison with high-dosage in-person rehabilitation.

**Trial Registration:**

ClinicalTrials.gov (NCT04636528); https://clinicaltrials.gov/study/NCT04636528

## Introduction

Chronic shoulder pain (CSP) is a common condition with various etiologies, including rotator cuff disorders, adhesive capsulitis, shoulder instability, and shoulder arthritis [1] with an annual prevalence of 16% [2]. It is one of the most frequent causes of ambulatory visits in the United States [3] and constitutes a significant socioeconomic burden on both patients and society, with one 2009 study estimating an annual cost of €4139 (US $5753) per patient [4].

CSP is associated with substantial disability and psychological distress [5], resulting in poor productivity and quality of life [5]. Surgical procedures are performed to treat CSP conditions [6], imposing an increased financial burden on individuals and society. More than 300,000 rotator cuff repairs were performed in the United States between 2007 and 2016 [7], with postsurgical costs reaching US $34,249 per patient [8]. Moreover, a significant proportion of patients will experience persistent postsurgical pain after surgery, with 1 study finding a rate of 35.6% (95% CI 26.1%-45.8%) in 101 patients who underwent arthroscopic subacromial decompression or acromioclavicular joint resection [9]. These observations contrast with evidence showing that exercise-based rehabilitation yields similar reductions in disability and pain outcomes compared to rotator cuff repair [10,11] and subacromial decompression [12,13]. Therefore, current recommendations prioritize nonsurgical approaches for CSP management through patient-centered multimodal care with exercise-based programs [14,15]. However, there are barriers to physical therapy (PT), including access to facilities and practitioners, long wait lists for appointments, geographic constraints, and transportation costs [16-18]. Further, the inability to schedule appointments outside of working hours and the burden of missing work may decrease adherence [16-18], ultimately compromising health outcomes [19].

Telerehabilitation gained momentum as a potential solution for the democratization of high-quality care, especially during the COVID-19 pandemic [20,21]. Telerehabilitation has shown numerous benefits, including improving access and convenience [20], promoting patient adherence [22], and reducing costs [23,24]. Growing evidence supports its safety and effectiveness in several musculoskeletal conditions compared to in-person interventions [22]. However, evidence on nonoperative CSP telerehabilitation remains insufficient [25], being based on small-sampled pilots [26-28], single-arm studies [29], or randomized controlled trials (RCT) comparing hybrid telerehabilitation (combining in-person and remote sessions) to in-person PT [30] or advice [31]. Previous studies have demonstrated the effectiveness of digital physical therapy (DGPT) programs that combine exercise, education, and cognitive behavioral therapy (CBT) to treat several musculoskeletal conditions [32,33] and to promote postsurgical rotator cuff recovery [34]. Its feasibility in addressing CSP has also been shown in a routine clinical context [29]. However, to date, no previous RCT has compared fully remote DGPT to in-person rehabilitation for nonoperative CSP.

The aim of this 8-week randomized controlled study is to compare clinical and self-reported health outcomes between DGPT and conventional in-person PT in patients with CSP. We hypothesized that outcomes would be similar in both interventions.

## Methods

### Ethics Approval

This single-center, parallel-group, randomized controlled study was approved by the University of California San Francisco (UCSF) Institutional Review Board (number 20-32636) and research ethics board and registered on ClinicalTrials.gov (NCT04636528) on November 19, 2020. Treatment and follow-up occurred between March 25, 2021, and December 15, 2022.

### Participants

Study participants were conveniently recruited from the UCSF outpatient practice and scheduled and screened for PT appointments. An in-person evaluation was conducted to ensure that potential candidates met the eligibility criteria. Participants provided written informed consent electronically through Castor eConsent (Castor Research Inc). All study-related information (including data from assessments) were stored in Castor electronic data capturing (EDC) platform, which is Food and Drug Administration (FDA) Code of Federal Regulations 21 Part 11 compliant.

The inclusion criteria were (1) patients between 18 and 80 years old; (2) intermittent or persistent tendon-related shoulder pain for at least 12 weeks, or at least 50% of the time in the past 6 months [35]; (3) the absence of visual, audio, or cognitive impairment interfering with the ability to understand or comply with the program. Exclusion criteria included (1) limited English proficiency; (2) residency outside of the greater San Francisco area; (3) known pregnancy; (4) surgery less than 3 months ago; (4) symptoms or signs indicative of possible infection; (5) referred pain from the spine or thoracic outlet syndrome; (6) active cancer diagnosis or undergoing treatment for cancer; (7) known disorder restricting tolerance to more than 20 minutes of light to moderate exercise; (8) concomitant neurological disorder that may interfere with participation or confound outcomes (eg, stroke, multiple sclerosis, or Parkinson disease); (9) known cognitive impairment, including dementia, psychiatric disorders, or other conditions (eg, visual or auditory impairment), or digital illiteracy precluding patient compliance with home-based exercise or interfering with communication; and (10) concurrent PT or another outside intervention for shoulder conditions during the study time. Exclusion criteria after eligibility screening (that included in-person evaluation) comprised missed assessment surveys, failure to start the intervention, and the development of a serious medical or psychosocial condition after screening but before enrollment [36,37]. Participants were considered dropouts if they (1) abandoned the study (ie, consent withdrawal) or (2) did not engage in any exercise session for 14 consecutive days in the DGPT group or missed 4 consecutive scheduled sessions in the conventional group (CG). Participants who were compliant with the intervention but failed to complete a given reassessment survey were included and analyzed as missing data.

### Allocation and Blinding

Randomization was performed by Castor EDC in a 1:1 ratio using random permuted blocks of 4-8 participants. Following randomization, allocation disclosure was conducted by the principal investigator, who communicated each participant’s allocation to respective investigators in each study arm, ensuring that blinding was maintained before allocation. Considering the nature of the intervention, investigators and participants were unblinded to group allocation. However, analyses were conducted independently by 2 statisticians blinded to group allocation.

### Intervention

#### Digital Intervention Group

**Figure 1:**
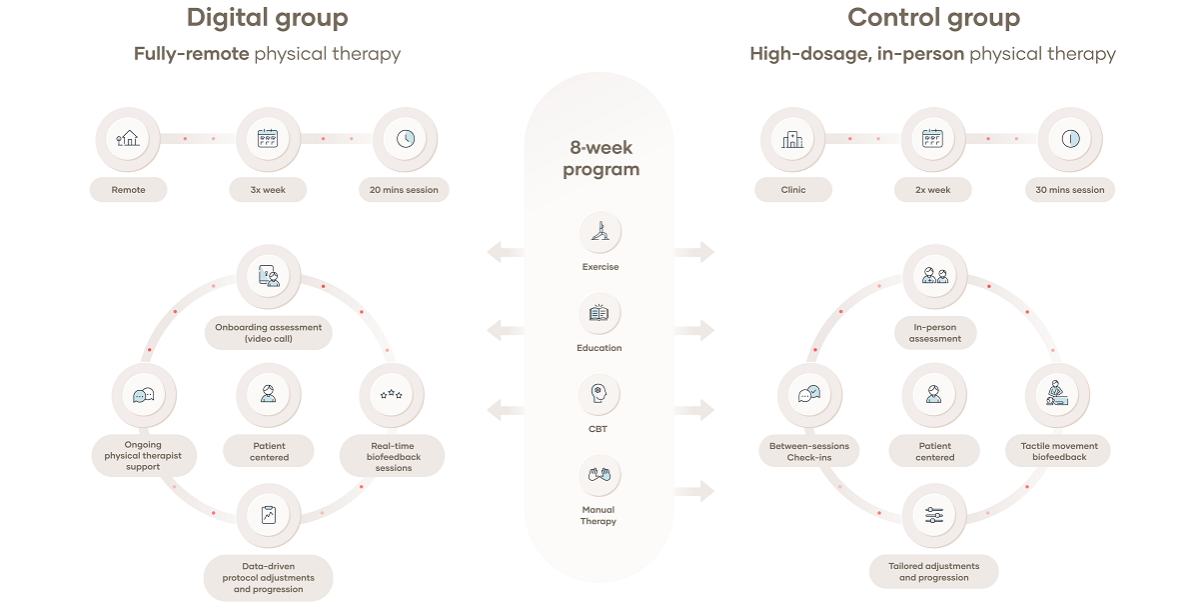
Schematic representation of the intervention for each group: the digital group (left side) and the control group (right side). CBT: cognitive behavioral therapy.

The digital intervention consisted of a home exercise program, patient education, and CBT (Figure 1), consistent with current guidelines [14,15]. Each participant was assigned and treated by a licensed physical therapist with clinical doctorate credentials. An initial onboarding video call consisted of a clinical evaluation, resulting in the prescription of an 8-week telerehabilitation program tailored to the participant’s needs. Asynchronous exercise sessions were performed independently at the participant’s convenience using an FDA-listed class II medical device consisting of 3 inertial motion trackers placed on the chest, upper arm, and wrist, a dedicated tablet with a mobile app, and a cloud-based portal (Figure S1 in Multimedia Appendix 1). The trackers provided movement quantification and digitalization to the mobile app, offering real-time biofeedback with video or audio cues that guided participants throughout exercise. Motion data were stored in the cloud-based portal with HITRUST and Google Cloud Platform’s Service Organization Control Type 2 (SOC2) certifications and were accessed and monitored asynchronously only by the physical therapist assigned to each participant, who adjusted the program accordingly. Personal health information was individually encrypted inside this database. Overall, 3 physical therapists were involved in the study with 13 years of experience on average (range 6-23). It was recommended that participants perform three 20-minute exercise sessions per week (total of 24 sessions). The exercises consisted of gradual painful movement exposure, mobility, stretching, and strengthening (Table S1 in Multimedia Appendix 2).

Patient education was delivered through a smartphone app in the form of short written articles addressing anatomy, physiology, pain reconceptualization, active coping skills, exercise, and fear-avoidance behaviors [14,15]. These topics were also included in the CBT program, which was composed of digital content and sent to participants through email. This program combined mindfulness, acceptance, commitment therapy, and empathy-focused therapy adapted to a curriculum focused on chronic pain.

Participants and the physical therapist were able to communicate through a built-in secure chat within a smartphone app for text messages and video or phone calls conducted on-demand (which was also conducted to motivate patients and increase adherence).

Technical and IT support was provided to patients through several communication channels. When hardware issues could not be resolved remotely, the device was replaced.

#### Conventional Intervention Group

The conventional intervention was an 8-week program consisting of in-person PT performed in a UCSF outpatient clinic under the supervision of physical therapists with clinical doctorate credentialing and board-certified orthopedic clinical specialists. Overall, 5 physical therapists were involved in the study, presenting on average 5 years of experience (range 4-8). The intervention included therapeutic exercises (similar to the DGPT), but through tactile movement or activity modification and gradual exposure based on McClure’s staged approach for shoulder rehabilitation (Figure 1 and Table S1 in Multimedia Appendix 3) [38]. This staged approach provides a framework that systematically selects and adjusts treatment algorithms to assess intervention and decision-making in stages based on different shoulder pain presentations [38]. The treatment framework was complemented with patient-specific manual therapy (eg, joint mobilization, tissue manipulation, and passive range of motion), education (including topics on wellness, prevention, anatomy, and exercise modifications), motivational interviewing, and CBT when appropriate [39]. Two 30-minute sessions per week were prescribed (total of 14-16 sessions), allowing for a similar treatment dosage between 2 groups. In addition to face-to-face sessions, physical therapists communicated with participants through telephone or email check-ins when appropriate.

### Outcomes

Primary and secondary outcomes were collected at baseline, 4, and 8 weeks in the form of electronic self-reported questionnaires through Castor EDC (except for engagement metrics). Study participants received a stipend of US $75 after completing the study.

The primary outcome was the change in function and symptoms measured through the short-form of Disabilities of the Arm, Shoulder, and Hand questionnaire (QuickDASH) between baseline and 8 weeks [40].

Secondary outcomes comprised:

1. Self-reported pain level: 11-point Numerical Pain Rating Scale for average pain experienced in the past 7 days, and the worst and least pain experienced in the past 24 hours (0: no pain and 10: worst pain imaginable).
2. Intention to undergo surgery: “On a scale of 0 to 10, where 0 is not at all and 10 is extremely interested, how interested are you in undergoing shoulder surgery in the next 12 months?”
3. Analgesic consumption: “Are you taking any medication for your shoulder pain? Yes/No”; and opioid consumption: “If yes, are you taking opioids for your shoulder pain? Yes/No.” Opioid dosage was not systematically recorded across the entire study.
4. Mental health: Generalized Anxiety Disorder 7-item scale (GAD-7; range 0-21) to assess anxiety [41,42], and Patient Health Questionnaire 9-item (PHQ-9; range 0-27) to assess depression [42,43]. Higher scores correspond to more severe symptoms.
5. Engagement: assessed through (1) treatment dosage (ie, total time spent on exercise sessions in minutes); (2) adherence to exercise sessions; and (3) dropout rates. These data were automatically collected by the tablet app in the digital PT and manually recorded by the physical therapist in the CG. Additionally, the number of educational and CBT content pieces consulted and the number of contacts were automatically collected by the mobile app or email in the digital PT or manually recorded by the physical therapist in the CG.
6. Patient satisfaction: “On a scale from 0 to 10, how likely is it that you would recommend this intervention to a friend or neighbor?”

### Safety and Adverse Events

Participants from the CG performed in-person sessions under direct physical therapist supervision. In the DGPT, the physical therapist used adherence, the existence or absence of movement errors, the level of pain and fatigue registered during exercises, and communication to adjust sessions. Both participants and physical therapists were instructed to contact the study investigators in case of an adverse event (registered on the Castor EDC platform).

### Sample Size

The sample size estimation was based on the primary outcome, QuickDASH. In a sample of 57 patients with work-related shoulder tendon conditions undergoing Sword’s DGPT, the baseline QuickDASH mean and SD were 59 and 19 points, respectively. A minimal detectable difference of 11.2 points was selected based on the psychometric properties of the scale [44]. Considering a power of 80%, a 2-sided 0.05 significance level, and a 10% dropout rate, 82 patients would be necessary to detect an 11.2-point difference between the 2 groups. The enrollment period was extended to acknowledge the unpredictability brought by the COVID-19 pandemic. Recruitment ceased once 82 patients were randomized and started the intervention.

### Statistical Analysis

Analyses were conducted considering intention-to-treat (ITT) (ie, all participants starting the study) and per-protocol approaches (ie, including all study completers). Data distribution was analyzed using the Kolmogorov-Smirnov test, followed by the inspection of histograms and Q-Q plots. Differences between groups in baseline characteristics and engagement metrics were assessed by independent sample *t* test or Mann-Whitney *U* test for quantitative variables and the Fisher exact test for categorical variables.

Clinical outcomes data revealed a non-normal distribution (Table S1 in Multimedia Appendix 4). Logarithmic and Box-Cox transformations were performed; however, these transformations did not result in normally distributed data (data not shown). Therefore, a quantile mixed-effects model using a robust method on the medians was selected for the analysis instead of repeated measures ANOVA [45]. Missing data were dealt with multiple imputation by chained equations [46]. The imputation was performed by applying different seeds that offered equivalent results, alongside sensitivity analyses for missing values on the ITT population, thus confirming validity. Both 8-week end-scores and cumulative changes between baseline and 8 weeks were compared between groups. Cohen *d* effect sizes were calculated for function and pain by comparing pre- and postintervention scores between groups.

The odds of being a responder for QuickDASH were calculated using logistic regression analysis, considering a 33% minimal clinically important difference (MCID) [47,48]. A relative MCID was selected to account for possible floor effects exerted by an absolute MCID, especially in populations with low baseline scores [49].

All analyses were performed using 2-sided hypothesis tests with a significance level of 0.05. The robust mixed-effects model was coded using R (version 4.2.2, R Foundation for Statistical Computing), and all other analyses used SPSS (version 28.0, IBM Corp).

## Results

### Overview

**Figure 2:**
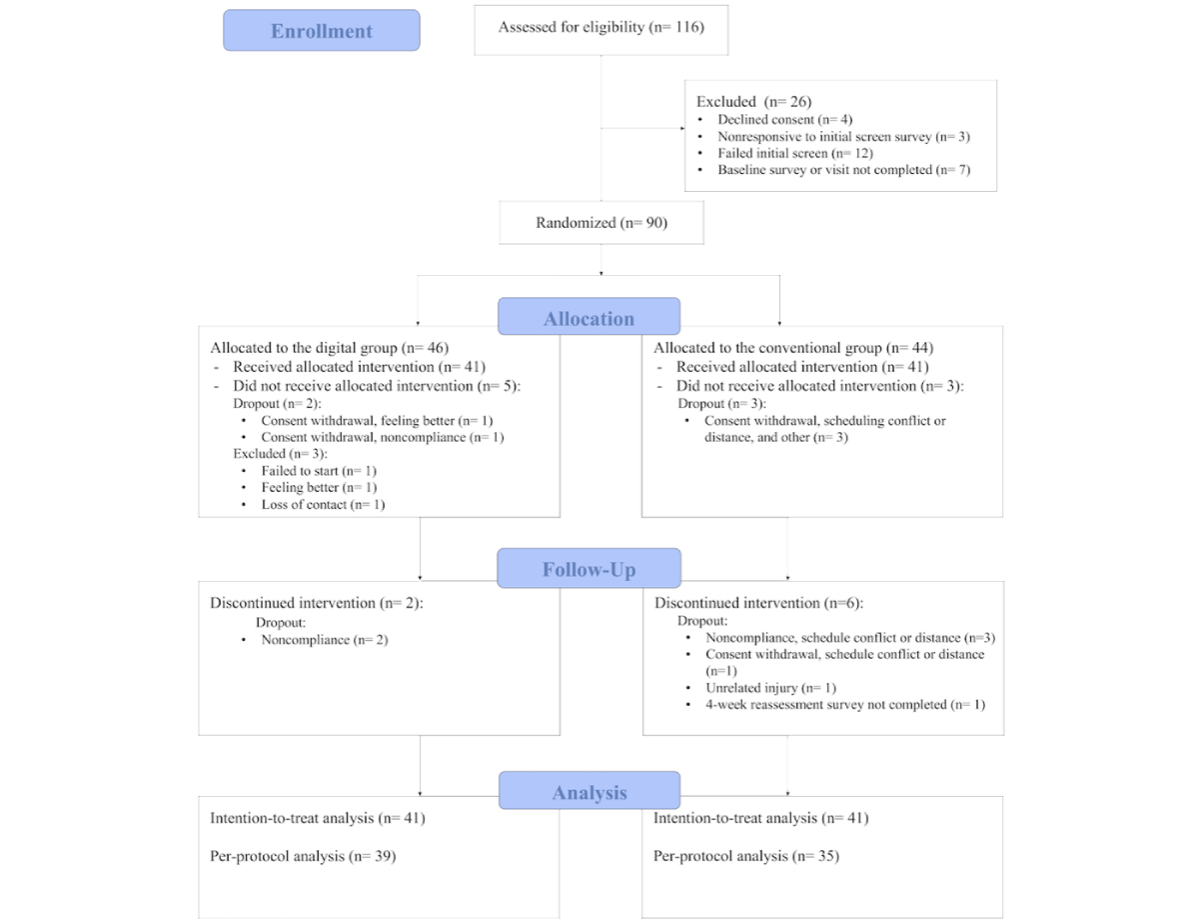
CONSORT (Consolidated Standards of Reporting Trials) flow diagram illustrating the participant flow throughout the study.

A total of 116 participants were screened for eligibility, of whom 26 (22.4%) were excluded, 4 (3.4%) declined consent, 15 (12.9%) failed to meet eligibility criteria, and 7 (6%) did not complete their baseline survey or initial visit (Figure 2). Overall, 90 participants were randomized either to the digital group (DGPT; n=46) or the CG (n=44), with 41 participants in each group receiving the allocated intervention. At study end, 84.8% (39/46) in the DGPT and 79.5% (35/44) in the CG had completed the intervention.

### Baseline Characteristics

There were no differences between groups in demographic characteristics or clinical scores using an ITT approach (N=82; Table 1), except for a higher least pain score in the CG (DGPT: median 1, IQR 2; and CG: median 2, IQR 3; Table 1).

Participants who did not complete the study (noncompleters, n=16) were not significantly different in terms of sociodemographics compared to program completers (n=74), except for a higher proportion of individuals with lower formal education and no participants from Asian or Pacific Islander backgrounds (Table S1 in Multimedia Appendix 5).

**Table 1:** Baseline characteristics of study participants (N=82).

Demographic characteristics		Digital group (n=41)	Conventional group (n=41)	*P* value^a^	
Age (years), mean (SD)		49.7 (12.6)	50.8 (12.9)	.71	
**Age categories (years), n (%)**					.96
	<25	1 (2.4)	0 (0)		
	25-40	11 (26.8)	10 (24.4)		
	40-60	17 (41.5)	19 (46.3)		
	>60	12 (29.3)	12 (29.3)		
**Gender, n (%)**					.27
	Woman	24 (58.5)	19 (46.3)		
	Man	16 (39)	22 (53.7)		
	Prefer not to answer	1 (2.4)	0 (0)		
BMI (kg/m^2^), median (IQR)		24.4 (5)	25.6 (7)	.27	
**BMI categories (kg/m^2^), n (%)**					.93
	Underweight (<18.5)	1 (2.4)	0 (0)		
	Normal (18.5-25)	22 (53.7)	20 (48.8)		
	Overweight (25-30)	12 (29.3)	13 (31.7)		
	Obese (30-40)	5 (12.2)	7 (17.1)		
	Morbidly obese (>40)	1 (2.4)	1 (2.4)		
**Race, n (%)**					.64
	Asian or Pacific Islander	14 (34.1)	9 (22)		
	Black or African American	1 (2.4)	2 (4.9)		
	Hispanic or Latino	3 (7.3)	2 (4.9)		
	White or Caucasian	17 (41.5)	24 (58.5)		
	Multiracial or biracial	5 (12.2)	3 (7.3)		
	Prefer not to answer	1 (2.4)	1 (2.4)		
**Education level, n (%)**					.34
	Some high school, GED^b^, or less	1 (2.4)	3 (7.3)		
	Some college or college degree	23 (56.1)	17 (41.5)		
	Some graduate or graduate degree	17 (41.5)	21 (51.2)		
**Employment status, n (%)**					.46
	Employed (part-time or full-time)	28 (68.3)	28 (68.3)		
	Unemployed (seeking opportunities)	6 (14.6)	4 (9.8)		
	Not employed and not seeking work	5 (12.2)	9 (22)		
	Prefer not to answer	2 (4.9)	0 (0)		
**Weekly exercise levels, n (%)**					.62
	None	1 (2.4)	0 (0)		
	Less than 1 hour	6 (14.6)	4 (9.8)		
	Between 1 and 2.5 hours	10 (24.4)	14 (34.1)		
	>2.5 hours	24 (58.5)	23 (56.1)		
**Comorbidities, n (%)**					
	High blood pressure	6 (14.6)	4 (9.8)	.74	
	High blood sugar or diabetes	2 (4.9)	3 (7.3)	>.99	
	Cardiac conditions	1 (2.4)	1 (2.4)	>.99	
	Respiratory conditions	3 (7.3)	1 (2.4)	.62	
	None of the above	38 (54.3)	37 (52.9)	.57	
Smoking habits, n (%)		1 (2.4)	1 (2.4)	>.99	
**Laterality of shoulder pain, n (%)**					.73
	Right	26 (63.4)	24 (58.5)		
	Left	13 (31.7)	16 (39)		
	Both	2 (4.9)	1 (2.4)		
Previous physical therapy, n (%)		18 (43.9)	13 (31.7)	.36	
Previous or scheduled shoulder surgery, n (%)		4 (9.8)	3 (7.3)	>.99	
**Clinical variables, median (IQR)**					
	QuickDASH^c^	25 (20.5)	25 (17.1)	.96	
	Pain level–worst	6 (4)	5 (4)	.52	
	Pain level–least	1 (2)	2 (3)	*.02*	
	Pain level–average	4 (3)	5 (3)	.28	
	Surgery intent	0 (4)	1 (15)	.16	
	GAD-7^d^	3 (4)	2 (6)	.53	
	PHQ-9^e^	3 (4)	2 (4)	.41	

^a^Mann-Whitney *U* test or Fisher exact test. Significant *P* values are presented in italics.

^b^GED: general educational development.

^c^QuickDASH: short-form of Disabilities of the Arm, Shoulder, and Hand questionnaire.

^d^GAD-7: Generalized Anxiety Disorder 7-item scale.

^e^PHQ-9: Patient Health Questionnaire 9-item.

### Clinical Outcomes

Outcomes following an ITT analysis are presented in Table 2 and respective model estimates are in Table S1 in Multimedia Appendix 6. Sensitivity analyses were performed, and the inspection of plots showed results robust to bias on missing data.

**Table 2:** Clinical outcomes at program end and respective differences between groups (end scores and total changes): intention-to-treat analysis (N=82).

Outcome variables	Digital group 8-week scores, median (95% CI)	Conventional group 8-week scores, median (95% CI)	Estimate 8-week scores difference between groups, median (95% CI)	*P* value	Estimate change difference between groups, median (95% CI)	*P* value
QuickDASH^a^	15.5 (7.7 to 23.2)	13.1 (5.8 to 20.3)	–2.3 (–14.7 to 10.0)	.71	–1.8 (–13.5 to 9.8)	.75
Pain level–worst	2.6 (2.2 to 3.0)	1.5 (1.2 to 1.8)	–0.9 (–1.6 to –0.3)	*.002* ^b^	–0.6 (0.2 to 1.4)	.12
Pain level–least	0.7 (0.6 to 0.7)	0.7 (0.5 to 0.7)	–0.1 (–0.2 to 0.0)	.15	–1.1 (–1.5 to 0.6)	<*.001*
Pain level–average	2.1 (1.9 to 2.3)	1.5 (1.4 to 1.7)	–0.6 (–0.9 to –0.4)	<*.001*	–0.9 (–1.4 to –0.5)	<*.001*
Surgery intent	6.3 (0.0 to 23.2)^c^	7.2 (0.0 to 25.1)^c^	1.4 (–33.2 to 36.0)	.94	–2.2 (–34.6 to 30.2)	.89
GAD-7^d^	2.8 (2.1 to 3.5)	2.2 (1.7 to 2.7)	–0.5 (–1.5 to 0.5)	.30	–0.3 (–1.0 to 0.5)	.51
PHQ-9^e^	2.7 (2.0 to 3.4)	1.6 (1.3 to 1.9)	–0.9 (–1.6 to –0.2)	<*.01*	–0.5 (–1.3 to 0.4)	.27

^a^QuickDASH: short-form of Disabilities of the Arm, Shoulder, and Hand questionnaire.

^b^Significant *P* values are presented in italics.

^c^CIs were fixed to 0 since analysis provided results outside the range of the corresponding scale.

^d^GAD-7: Generalized Anxiety Disorder 7-item scale.

^e^PHQ-9: Patient Health Questionnaire 9-item.

### Primary Outcome

**Figure 3:**
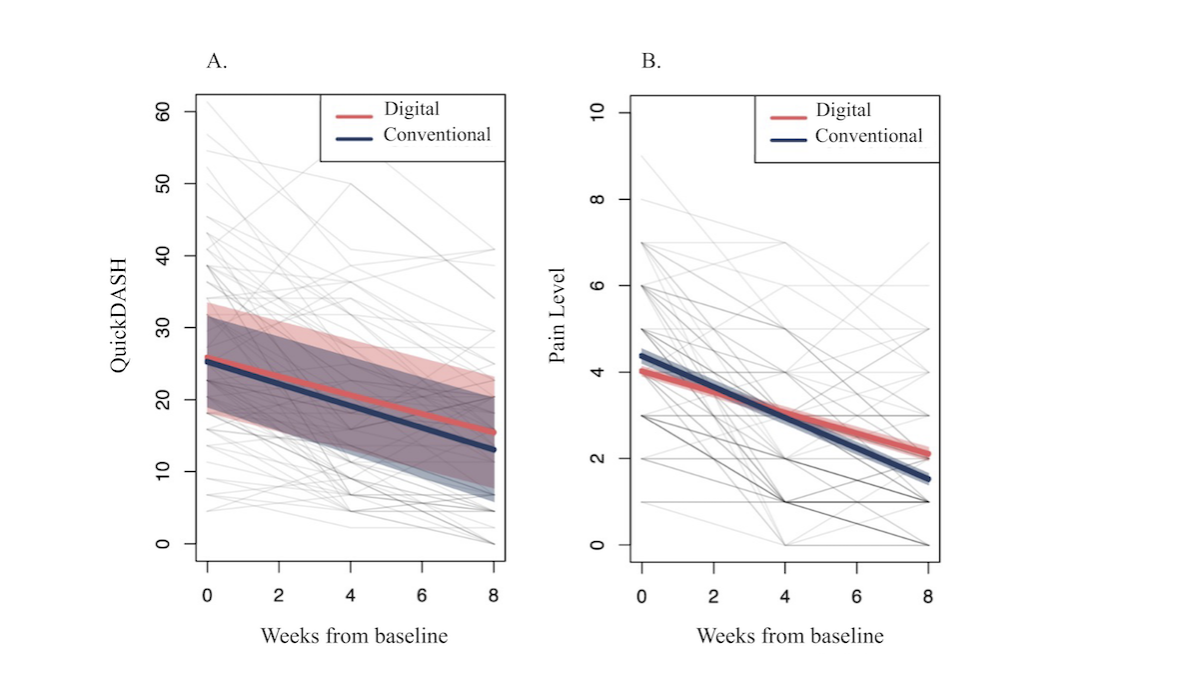
Longitudinal changes across time for (A) short-form of Disabilities of the Arm, Shoulder, and Hand questionnaire (QuickDASH) and (B) average pain until intervention-end per group. The colored lines depict the mean trajectories for each group, with shadowing representing CIs. Individual trajectories are depicted in light gray lines.

Significant improvements in function were observed after both interventions (DGPT: median –10.4, 95% CI –17.0 to –3.7 and CG: median –11.8, 95% CI –19.1 to –4.6; both groups *P*=.002). The QuickDASH change between baseline and 8 weeks was similar between groups (difference: median –1.8, 95% CI –13.5 to 9.8; *P*=.75), corresponding to an effect size of 0.01. Both groups ended with comparable scores (DGPT: median 15.5, 95% CI 7.7-23.2 and CG: median 13.1, 95% CI 5.8-20.3; *P*=.71; Figure 3 and Table 2). The response rate, defined as an MCID >33%, was similar between groups (DGPT: 24/39, 61.5% and CG: 23/35, 65.7%; odds ratio 0.84, 95% CI 0.32-2.16; *P*=.71; reference: CG).

### Secondary Outcomes

#### Pain

Each group reported significant reductions in the 3 pain outcome measures (eg, pain average: DGPT: median –2.0, 95% CI –2.2 to –1.8 and CG: median –2.9, 95% CI –3.2 to –2.6; both *P*<.001). The reduction in average pain was statistically different between groups (median –0.9, 95% CI 0.5-1.4; *P*<.001). Participants in the CG attained a higher change in least pain (median –1.1, 95% CI –1.5 to 0.6; *P*<.001); however, they started with higher baseline scores (DGPT: median 1, SD 2 and CG: median 2, SD 3; *P*=.02). No significant difference in worst pain was observed between groups (median –0.6, 95% CI 0.2-1.4; *P*=.12). Small effect sizes were obtained when comparing the between-group changes in pain variables (worst pain: 0.08; least pain: 0.15; and average pain: 0.16) suggesting the absence of a clinically meaningful difference [50].

#### Intention to Undergo Surgery

Participants reported low levels of intention to pursue surgery at baseline (DGPT: median 0, IQR 4 and CG: median 1, IQR 15; Table 1). No significant within-group changes were observed in either intervention (DGPT: median –1.6, 95% CI –13.4 to 10.2; *P*=.83 and CG: median –2.7, 95% CI –22.7 to 17.3; *P*=.79), with both groups reporting similar end scores and overall changes (Table 2).

#### Analgesic Consumption

The difference between groups in baseline levels of analgesic intake was not statistically significant: 4.9% (2/41) participants in the DGPT and 17.1% (7/41) in the CG reported taking analgesics (*P*=.16). Among those participants taking analgesics at baseline, only 1 participant in the CG reported taking opioids.

#### Mental Health

Significant and similar reductions in depression symptoms were observed in both groups from differences of estimated change (within-group changes: DGPT: –0.6, 95% CI –1.0 to –0.2 and CG: –1.1, 95% CI –1.6 to –0.6; both *P*<.001; overall change difference: –0.5, 95% CI –1.3 to 0.4; *P*=.27; Table 2). Regarding anxiety, no significant within-group changes were observed in either group (DGPT: –0.3, 95% CI –0.6 to 0; *P*=.06 and CG: –0.4, 95% CI –0.8 to 0.1; *P*=.09), with both groups experiencing similar end-of-treatment scores (Table 2).

### Patient Engagement

Overall, participants were exposed to a similar treatment dosage in both groups (Table 3). A total of 26.1 (SD 11.6) exercise sessions were performed in the DGPT, while participants in the CG completed 13.4 (SD 4) in-person sessions. The dropout rate was slightly lower in the DGPT compared to the CG (DGPT: 4/46, 8.7% and CG: 9/44, 20.5%; *P*=.14), but not significant.

Engagement with education and behavioral change components was delivered digitally in a scheduled format for the DGPT and verbally in person “as needed” in the CG. The DGPT read a median of 4 (IQR 4.3) educational articles and engaged with a median of 9 (IQR 6) CBT content pieces. The CG administered education components a median of 8 (IQR 6) times, with specific CBT-focused components in 8 subjects.

Satisfaction with the program was high in both groups, with participants from the CG reporting higher levels of satisfaction (CG: median 10, IQR 1 and DGPT: median 8, IQR 5; *P*<.001).

**Table 3:** Engagement metrics of participants.

Engagement variable		Participants, n	Digital group, mean (SD)	Participants, n	Conventional group, mean (SD)	*P* value^a^	
**Total time during sessions (minutes)**							
	ITT^b^	41	461.6 (218.2)	41	393.9 (118.8)	.36	
	PP^c^	39	481.6 (204)	35	430.9 (60.6)	.75	
**Total sessions**							
	ITT	41	26.1 (11.6)	41	13.4 (4)	<.001	
	PP	39	27.2 (10.7)	35	14.7 (2)	<.001	
**Frequency of sessions per week**							
	ITT	41	3.3 (1.4)	41	1.68 (0.4)	<.001	
	PP	39	3.4 (1.3)	35	1.86 (0.3)	<.001	

^a^Mann-Whitney *U* test.

^b^ITT: intention-to-treat.

^c^PP: per-protocol.

### Safety and Adverse Events

There were no adverse events related to the study (either unexpected, definitely, probably, or possibly research-related).

## Discussion

### Principal Findings

The purpose of this study was to compare the clinical outcomes between DGPT and conventional in-person PT for patients with CSP. For the primary outcome (ie, QuickDASH), we observed a statistically significant change within each group and no difference between groups, along with a similar proportion of treatment responders. For secondary outcomes, no differences were observed in surgery intent, analgesic intake, and anxiety between groups, and for these outcomes, within-group pre-post changes were not significant. Similar improvements in depression symptoms were noted in both groups. Higher reductions were observed in average and least pain in the CG but not in the worst pain, which were unlikely to be clinically meaningful given the small effect sizes (worst pain: 0.08; least pain: 0.15; and average pain: 0.16). Both groups underwent similar treatment dosage and reported high satisfaction scores, low dropout rates, and no adverse events, denoting the acceptance and safety of both care delivery models. Overall, this study demonstrates for the first time the effectiveness of fully remote DGPT in patients with nonoperative CSP compared to in-person PT. These findings suggest that the delivery of PT through digital interventions can be an effective care pathway for patients with CSP.

### Comparison With Literature

#### Acceptance and Engagement

This study showed low dropouts and high satisfaction in both groups, with the CG reporting higher scores than DGPT (CG: median 10, IQR 1 and DGPT: median 8, IQR 5; *P*<.001). These findings suggest patient’s acceptance of interventions, although not all domains that constitute acceptance were evaluated. This is in line with previous research assessing patients’ perspectives and perceptions of telerehabilitation in qualitative studies [51,52]. Telerehabilitation has historically been associated with similar or lower dropout rates than in-person PT [24,25,53]. Herein, high completion rates were observed across groups, within the range of those previously reported in previous trials assessing exercise-based conservative interventions in shoulder conditions [54-56]. The DGPT reported a slightly lower dropout rate than the CG (8.7% vs 20.5%; *P*=.11), a trend previously reported in nonoperative CSP [30]. A potential contributor to the higher dropout rate in CG may have been the surge of the COVID-19 pandemic in San Francisco County during the study period [57].

Adherence to PT is key to improving outcomes and preventing treatment escalation [16,19,58]. Telerehabilitation has the potential to improve adherence through increased accessibility [59], convenience, and flexibility to fit daily routines [22]. Engagement with remote care can be enhanced through functionalities such as continued remote monitoring, exercise biofeedback, gamification, and accessible communication (between patients and physical therapists through video calls, phone calls, or messages) [19,60-62]. The incorporation of these in the present DGPT may have contributed to the high engagement.

#### Clinical Outcomes

High-quality evidence for the effectiveness of hybrid or remote telerehabilitation solutions for shoulder conditions is still scarce, as denoted by a recent systematic review [25] that reported no differences in functional improvement between telerehabilitation and in-person PT (effect sizes 0.60, 95% CI –0.32 to 1.53). Herein, similar results were obtained between in-person and digital interventions. Both groups reported comparable functional improvements and similar response rates. Similarly, significant within-group pain reductions were observed in both groups. The reduction in average pain was statistically different between groups but probably not clinically meaningful given the small effect sizes between groups.

#### Mental Health

The 2-way feedback loop between mental health, pain, and disability is widely acknowledged [63] and is reflected in the paradigm shift toward a biopsychosocial approach for musculoskeletal pain management [14,15]. Education and behavioral change interventions are pivotal components of a holistic program [19,61,64]. Despite their inclusion in many multimodal in-person interventions, most studies within telerehabilitation only provide exercise [25,65]. Although no significant impact was observed in anxiety in either group, significant and comparable improvements in depression symptoms were reported for both interventions. Whereas psychological outcomes are infrequently evaluated in telerehabilitation trials, there is growing evidence that these interventions can positively impact the mental health domain [66-68].

#### Analgesic Intake and Willingness to Undergo Surgery

The prevalence of analgesic intake for CSP varies across the literature, with some studies demonstrating very high levels (80%-100%) [31,69], while others report values in line with this study [26]. PT can contribute to a reduction in analgesic intake [70,71]. In this study, the low proportion of patients receiving pharmacological treatment at baseline (DGPT: 2/41, 4.9% and CG: 7/41, 17.1%) limited the statistical assessment of this aspect. Analgesic intake has been poorly investigated in research focused on nonoperative CSP [70], a gap that should be addressed in future trials.

Surgery intent at baseline was very low, which translated to a lack of significant within-group changes in either group. This might be related to the PT referral process, which involves initial physician consultation to potentially exclude individuals with poor clinical presentations and imaging supportive of surgery referral. Additionally, the referral process and educational process may have influenced baseline and end-of-treatment surgery intent, respectively, by anchoring participants to the potential benefits of an exercise intervention.

### Strengths and Limitations

The major strength of this work is the study design: a preregistered RCT with an active comparator group consisting of high-dosage, in-person PT with similar treatment dosage between groups, facilitating comparison of outcomes. The novelty of the digital intervention is another strength, as a fully remote exercise-based PT, supported by real-time biofeedback and embedded in a multimodal intervention including education and CBT. The remote monitoring and ongoing contact with the physical therapist through synchronous and asynchronous communications may also have contributed to the high adherence. Incorporating standardized self-reported outcome measures to assess different health domains represents an additional strength of the study [40-43]. Contrary to most current literature, where engagement measures lack standardization and are often reported using subjective metrics, this study used objective metrics that foster future comparisons [72].

There are several limitations to this study that warrant discussion. First, this study was undertaken in the context of the COVID-19 pandemic, which may have impacted perceptions, receptivity, and compliance with digital and in-person PT programs. Second, the enrolled cohort was composed of predominantly young, physically active, highly educated individuals residing in urban areas, which may limit generalizability. Third, a relatively small proportion of these individuals were receiving regular analgesic medications and were considering surgery, which again merits caution with extrapolation. Fourth, administration of the CBT and educational components varied between the 2 groups, even if they were similar in concept. Fifth, both investigators and participants were unblinded to group allocation. Last, this study lacked posttreatment follow-up, which prohibits conclusions on long-term benefit and cost-effectiveness. Future research should address these issues, endeavor to identify factors that may influence an individual’s decision to partake in digital, in-person, or hybrid care, and define criteria for appropriate candidate selection. The adoption of these new models of care in routine clinical practice also requires competencies by physical therapists, including important changes in training curricula.

### Conclusions

This study shows digital programs can be viable care delivery models for CSP. High acceptance, satisfaction, and adherence rates were observed in both groups, along with significant and comparable improvements in clinical outcomes. These findings highlight the potential of digital care pathways to deliver PT to ease the current burden of CSP, considering their capacity for scalability, viability, and effectiveness.

## Appendix

Multimedia Appendix 1 Representation of the digital device. (A) Kit composed by a dedicated tablet with the app and motion trackers. (B) cloud-based portal accessed and monitored by the physical therapist. (C) Exercise execution display on the tablet during sessions with real-time visual biofeedback.

Multimedia Appendix 2 Description of the exercise prescription in the digital group.

Multimedia Appendix 3 Description of interventions in the conventional group.

Multimedia Appendix 4 Tests of normality.

Multimedia Appendix 5 Baseline characteristics between study completers (n=74) and noncompleters (n=16).

Multimedia Appendix 6 Clinical outcomes estimates by the quantile mixed-effects model: intention-to-treat analysis (N=82).

Multimedia Appendix 7 CONSORT-EHEALTH checklist (V 1.6.1).

## Abbreviations

CBT: cognitive behavioral therapy
CG: conventional group
CSP: chronic shoulder pain
DGPT: digital physical therapy
EDC: electronic data capturing
FDA: Food and Drug Administration
GAD-7: Generalized Anxiety Disorder 7-item
ITT: intention-to-treat
MCID: minimal clinically important difference
PHQ-9: Patient Health Questionnaire 9-item
PT: physical therapy
QuickDASH: short-form of Disabilities of the Arm, Shoulder, and Hand questionnaire
RCT: randomized controlled trial
SOC2: Service Organization Control Type 2
UCSF: University of California San Francisco

## Data Availability

All relevant data underlying the study are included in the article or available as supplementary material. Raw data and statistical codes will be made available upon request. The protocol of the study and corresponding results will be available on ClinicalTrials.gov upon publication for at least 5 years.
