# Supplementary figures and images for: Comparing Digital to Conventional Physical Therapy for Chronic Shoulder Pain: Randomized Controlled Trial
Source: J Med Internet Res. 2023 Aug 18;25:e49236. doi: 10.2196/49236 (PMC10474513; doi:10.2196/49236)

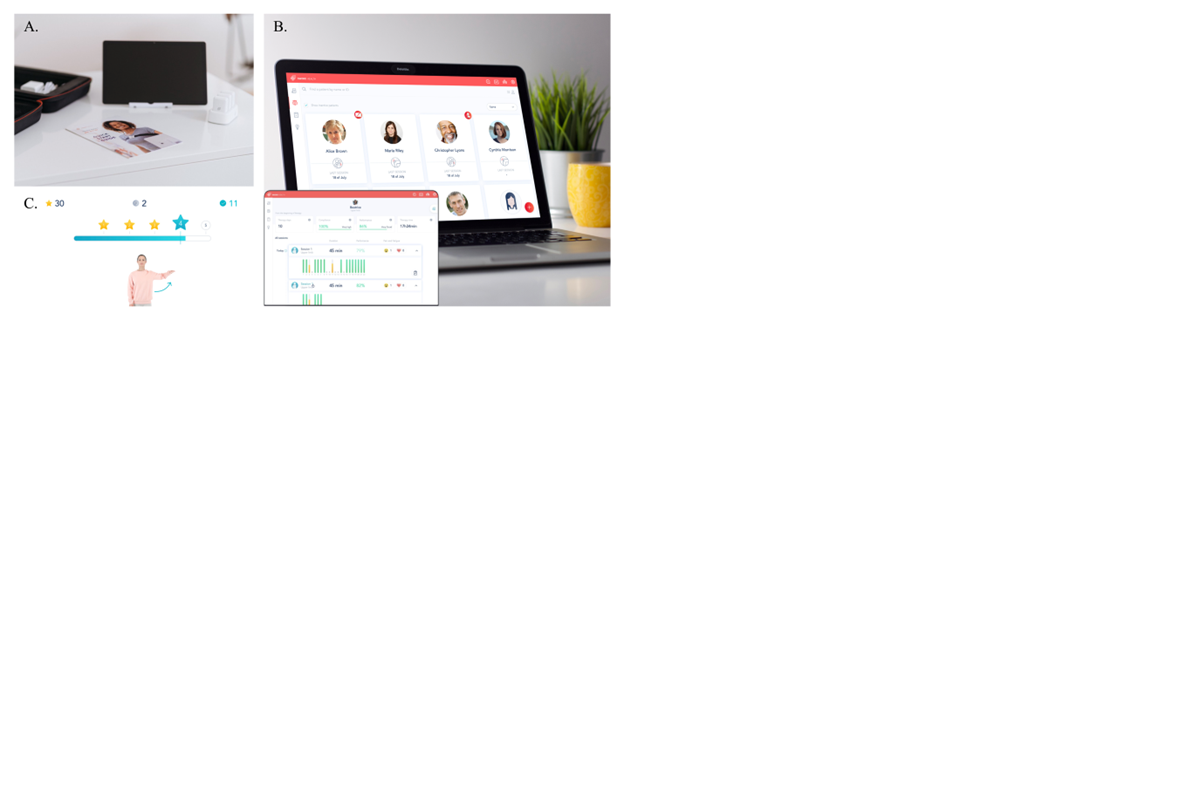

Supplement: Multimedia Appendix 1 [file jmir_v25i1e49236_app1.png]
