# Supplementary material for: Comparing Digital to Conventional Physical Therapy for Chronic Shoulder Pain: Randomized Controlled Trial
Source: J Med Internet Res. 2023 Aug 18;25:e49236. doi: 10.2196/49236 (PMC10474513; doi:10.2196/49236)
Supplement: Multimedia Appendix 2 [file jmir_v25i1e49236_app2.docx]

# **Table S1.** Description of the exercise prescription in the digital group.

| **Phases and Main goals** | **Intervention description** |
| --- | --- |
| **Early Phase**  **Goals:**   - Reduction of symptom intensity/severity   (eg. pain and paresthesia) and inflammatory signs   - Improve towards full range of motion (passive and active) - Improve muscular strength - Advocate use of unaffected regions | Exercises are based on assessment of symptom irritability and gradual exposure to movements and activities that elicit symptoms, comprising:   - At least 2 sets of upper limb and scapula mobility and stretching exercises - At least 2 sets of strengthening exercises targeting the rotator cuff, scapular stabilizers and primary mover muscles, using external load through bands or free weights or bodyweight.     These include:   - Uni-joint (ie. exercises involving movement of one single joint. E.g., standing shoulder flexion and lying shoulder external rotation) and multi-joint movement (ie. exercises involving movement of at least two joints, also named compound movements. E.g., incline push-ups and standing row) exercises. - Isometric and dynamic exercises |
| **Late Phase**  **Goals:**   - Maintain full range of motion - Increase global muscular strength and endurance - Increase time of exposure to upper limb movements - Return to all activities of daily living - Foster independent symptom management - Decrease fear-avoidance of movement | The late phase , interventions include the following with progressions from early phrase exercises:   - Increase the range of motion of the prescribed exercises - Increase load: increase external load on free weights or band resistance or increase the load on bodyweight exercises (e.g. decrease height of incline push ups) - Increase average session time (increasing sets and/or repetitions of the prescribed exercises) |
| ***Note:*** *The exercise prescription and subsequent adjustments during the intervention were based on the initial evaluation and individual patient progress and performance during the program.*  *The parameters of each strengthening and range of motion exercise were adjusted and progressed, namely: range of motion, number of exercises, number of sets and repetitions.*  *Besides ongoing patient’s assessment and communication, additional feedback from exercises was monitored based on the following parameters: movement errors, plateau effect and self-reported pain and fatigue.*  *Precautions include: avoiding exercises that exacerbate pain over 5/10; restrict range of motion on flexion and abduction movements in exercises with external load in the initial stage; restricting push-ups on the floor in case of complete rotator cuff tears (use inclined planes instead).* | |
