# Supplementary material for: Comparing Digital to Conventional Physical Therapy for Chronic Shoulder Pain: Randomized Controlled Trial
Source: J Med Internet Res. 2023 Aug 18;25:e49236. doi: 10.2196/49236 (PMC10474513; doi:10.2196/49236)
Supplement: Multimedia Appendix 3 [file jmir_v25i1e49236_app3.docx]

**Table S2.** Description of interventions in the conventional group.

| **Stages and Main Goals** | **Intervention Description** |
| --- | --- |
| **Early Phase**  **Goals:**   - Restore passive/active assist range of motion - Improve muscular strength - Activity modification - Basic-level functional activity restoration - Minimize physical stress | Interventions including exercises and tactile cues that are based on assessment of irritability symptoms and mobility of tissue. Examples:   - Isometrics (e.g., ER/IR, scapular squeezes) - Active and passive stretches (e.g., gentle sleeper stretches; Foam roller pec stretches/ snow angels; modified to ½ to full foam roller ; Modified doorway stretch) - Mirror/manual cue feedback for AROM/AAROM; Scapular clock exercises |
| **Late Phase**  **Goals:**   - Full restoration of range of motion - Improve muscular strengths - Improve neuromuscular weakness associated with poor motor control or neural activation - Gradual to full return to activity of daily living - Improve overall conditions and wellness | Interventions, including exercises and tactile cues are based on assessment of moderate to low irritability symptoms and mobility of tissue. Examples:   - Isometrics to isokinetic to dynamic resistive (e.g., ER/IR, rows, prone I’s, T’s Y’s) - Rotator cuff strengthening (ER/IR/Flexion/Abduction) in progression from neutral to variations of movements via weights or T-band - Progress graded activities and high-demand movement training with emphasis on quality rather than resistance according to motor learning principles - Loaded motor control exercises with mirror feedback and closed Kinetic Chain Upper Extremity Exercises (plank, push ups) - Aggressive active and passive stretches (e.g., sleeper stretches; foam roller pec and upper thoracic mobility; Doorway stretch, Contract Relax stretching) |
| ***Note:*** *The exercise prescription and subsequent adjustments during the intervention were based on the initial evaluation and individual patient progress and performance during the program, and, additionally, used McClure's staged based rehabilitation therapy (STAR) to guide prescription. The STAR approach provides a framework to adjust and progress the intervention according to the irritability of the patient's condition through the various stages that can be accommodated through in-person, tactile assessments. Participants were instructed to continue to perform the exercises at home.*  *Precautions include worsening of symptoms during and post interventions; avoid aggressive active and passive stretches for high irritable symptoms in clinical presentations.*  *Abbreviations: AAROM, active assisted range of motion; AROM, active range of motion; ER, external rotation; IR, internal rotation.* | |
