# Supplementary material for: Comparing Digital to Conventional Physical Therapy for Chronic Shoulder Pain: Randomized Controlled Trial
Source: J Med Internet Res. 2023 Aug 18;25:e49236. doi: 10.2196/49236 (PMC10474513; doi:10.2196/49236)
Supplement: Multimedia Appendix 4 [file jmir_v25i1e49236_app4.docx]

Table S3

| **Tests of Normality** | | | | | | | |
| --- | --- | --- | --- | --- | --- | --- | --- |
|  | Group | Kolmogorov-Smirnov^a^ | | | Shapiro-Wilk | | |
|  |  | Statistic | df | Sig. | Statistic | df | Sig. |
| QuickDASH scores  at baseline | Conventional | .167 | 35 | *.02* | .958 | 35 | .20 |
|  | Digital | .153 | 39 | *.02* | .956 | 39 | .13 |
| QuickDASH scores  at 8-weeks | Conventional | .184 | 35 | *.004* | .921 | 35 | *.02* |
|  | Digital | .125 | 39 | .13 | .945 | 39 | .06 |
| a. Lilliefors Significance Correction  Significant p-values are presented in italic. | | | | | | | |

|  | Group | Kolmogorov-Smirnov^a^ | | | Shapiro-Wilk | | |
| --- | --- | --- | --- | --- | --- | --- | --- |
|  |  | Statistic | df | Sig. | Statistic | df | Sig. |
| Pain scores at baseline | Conventional | .123 | 35 | .20 | .969 | 35 | .41 |
|  | Digital | .186 | 39 | *.002* | .935 | 39 | *.03* |
| Pain scores at 8-weeks | Conventional | .255 | 35 | *<.001* | .848 | 35 | *<.001* |
|  | Digital | .190 | 39 | *.001* | .902 | 39 | *.002* |
| a. Lilliefors Significance Correction  Significant p-values are presented in italic. | | | | | | | |
