# Supplementary material for: Comparing Digital to Conventional Physical Therapy for Chronic Shoulder Pain: Randomized Controlled Trial
Source: J Med Internet Res. 2023 Aug 18;25:e49236. doi: 10.2196/49236 (PMC10474513; doi:10.2196/49236)
Supplement: Multimedia Appendix 5 [file jmir_v25i1e49236_app5.docx]

**Table S4.** Baseline characteristics between study completers (N=74) and non-completers (N=16).

| **Characteristic** | **Completers**  **(N=74)** | **Non-completers**  **(N=16)** | ***P*a** |
| --- | --- | --- | --- |
| *Age (years), median (IQR*) | 50.5 (12.7) | 48.9 (16.4) | .87^b^ |
| *Age categories, N (%):* |  |  | .95 |
| <25 | 1 (1) | 0 (0) |  |
| 25-40 | 19 (26) | 5 (31) |  |
| 40-60 | 31 (42) | 6 (38) |  |
| > 60 | 23 (31) | 5 (31) |  |
| *Gender, N (%):* |  |  | 0.99 |
| Woman | 39 (53) | 8 (50) |  |
| Man | 34 (46) | 8 (50) |  |
| Prefer not to answer | 1 (1) | 0 (0) |  |
| *BMI, median (IQR)* | 24.4 (6.0) | 25.9 (4.0) | .20 |
| *BMI categories, N (%):* |  |  |  |
| Underweight (<18.5) | 1 (1) | 0 (0) | .19 |
| Normal (18.5-25) | 41 (55) | 5 (31) |  |
| Overweight (25-30) | 21 (28) | 8 (50) |  |
| Obese (30-40) | 10 (14) | 2 (13) |  |
| Morbidly obese (>40) | 1 (1) | 1 (6) |  |
| *Race, N (%):* |  |  | *.01* |
| Asian or Pacific Islander | 23 (31) | 0 (0) |  |
| Black or African American | 2 (3) | 1 (6) |  |
| Hispanic or Latino | 4 (5) | 2 (12) |  |
| White or Caucasian | 37 (50) | 10 (62) |  |
| Multi-racial or biracial | 7 (10) | 1 (6) |  |
| Prefer not to answer | 1 (1) | 2 (12) |  |
| *Education level, N (%):* |  |  | *.02* |
| Some high school/GED or less | 1 (1) | 3 (19) |  |
| Some college/college degree | 36 (49) | 8 (50) |  |
| Some graduate or graduate degree | 27 (50) | 5 (31) |  |
| *Employment status, N (%):* |  |  | .92 |
| Employed (part-time or full-time) | 50 (68) | 10 (62) |  |
| Unemployed (seeking opportunities) | 9 (12) | 2 (12) |  |
| Not employed | 13 (18) | 4 (25) |  |
| Prefer not to answer | 2 (3) | 0 (0) |  |
| *Weekly exercise levels, N (%):* |  |  | .19 |
| None | 0 (0) | 1 (6) |  |
| Less than 1 hour | 8 (11) | 2 (12) |  |
| Between 1-2.5 hours | 22 (30) | 6 (38) |  |
| >2.5 hours | 44 (60) | 7 (44) |  |
| *Comorbidities, N (%):* |  |  |  |
| High blood pressure | 10 (14) | 0 (0) | .20 |
| High blood sugar or diabetes | 5 (7) | 0 (0) | .58 |
| Cardiac conditions | 2 (3) | 1 (6) | .45 |
| Respiratory conditions | 3 (4) | 2 (12) | .22 |
| None of the above | 60 (81) | 13 (81) | .99 |
| *Smoking habits, N (%):* | 1 (1) | 1 (6) | .33 |
| *Laterality of shoulder pain, N (%):* |  |  | .99 |
| Right | 44 (60) | 10 (62) |  |
| Left | 27 (36) | 6 (38) |  |
| Both | 3 (4) | 0 (0) |  |
| *Previous physical therapy, N (%):* | 28 (38) | 5 (31) | .78 |
| *Previous or scheduled shoulder surgery, N (%):* | 5 (7) | 3 (19) | .15 |
| ^a^Mann–Whitney U test or Fisher’s exact test; ^b^independent samples t-test  Significant p-values are presented in italic. | | | |
