# Supplementary material for: Comparing Digital to Conventional Physical Therapy for Chronic Shoulder Pain: Randomized Controlled Trial
Source: J Med Internet Res. 2023 Aug 18;25:e49236. doi: 10.2196/49236 (PMC10474513; doi:10.2196/49236)
Supplement: Multimedia Appendix 6 [file jmir_v25i1e49236_app6.docx]

# **Table S5.** Clinical outcomes estimates by the quantile mixed-effects model: intention-to-treat analysis (N=82).

| **Outcome Variables,**  **Median (95% CI)** | **Digital Group** | | | | **Conventional Group** | | | |
| --- | --- | --- | --- | --- | --- | --- | --- | --- |
|  | **N** | **Intercept** | **Slope** | ***P*** | **N** | **Intercept** | **Slope** | ***P*** |
| **QuickDASH** | 41 | 25.9 (16.8 to 34.9) | -1.3 (-1.4 to -1.1) | <.001 | 41 | 25.3 (18.3 to 32.3) | -1.5 (-1.6 to -1.4) | <.001 |
| **Pain Level - Worst** | 41 | 5.0 (4.8 to 5.2) | -0.5 (-0.5 to -0.4) | <.001 | 41 | 5.3 (5.1 to 5.6) | -0.4 (-0.4 to -0.4) | <.001 |
| **Pain Level - Least** | 41 | 2.0 (1.9 to 2.2) | -0.2 (-0.2 to -0.2) | <.001 | 41 | 1.3 (1.2 to 1.5) | -0.1 (-0.1 to -0.1) | <.001 |
| **Pain Level - Average** | 41 | 4.0 (3.9 to 4.1) | -0.3 (-0.2 to -0.3) | <.001 | 41 | 4.4 (4.2 to 4.5) | -0.4 (-0.4 to -0.4) | <.001 |
| **Surgery Intent** | 41 | 7.7 (0.0 to 24.0)^a^ | -0.9 (-1.2 to -0.7) | <.001 | 41 | 9.2 (0.0 to 22.9)^a^ | -1.1 (-1.3 to -0.9) | <.001 |
| **GAD-7** | 41 | 3.3 (2.8 to 3.9) | -0.1 (-0.1 to -0.1) | <.001 | 41 | 3.1 (2.2 to 4.0) | -0.1 (-0.1 to -0.1) | <.001 |
| **PHQ-9** | 41 | 3.5 (3.0 to 3.9) | -0.2 (-0.2 to -0.2) | <.001 | 41 | 2.9 (2.4 to 3.3) | -0.2 (-0.2 to -0.2) | <.001 |
| **Abbreviations:** GAD-7, Generalized Anxiety Disorder 7-item scale; PHQ-9, Patient Health 9-item questionnaire; QuickDASH, Quick Disabilities of the Arm, Shoulder and Hand questionnaire.  ^a^Confidence intervals were fixed to zero because analysis provided results outside the range of the corresponding scale. | | | | | | | | |
